# Sodium hydroxide treatment effectively inhibits PrP^CWD^ replication in farm soil

**DOI:** 10.1080/19336896.2019.1617623

**Published:** 2019-07-01

**Authors:** Hyun-Joo Sohn, Kyung-Je Park, In-Soon Roh, Hyo-Jin Kim, Hoo-Chang Park, Hae-Eun Kang

**Affiliations:** Foreign Animal Disease Division, Department of Animal and Plant Health Research, Animal and Plant Quarantine Agency, Gimcheon-si, Gyeongsangbukdo, Republic of Korea

**Keywords:** CWD, farm soil, 2N NaOH, NaClO, TgElk, PMCAb

## Abstract

Chronic wasting disease (CWD) agents are shed into biological samples, facilitating their horizontal transmission between cervid species. Once prions enter the environment, binding of PrP^CWD^ by soil particles may maintain them near the soil surface, posing a challenge for decontamination. A 2 N sodium hydroxide (NaOH) or 2% sodium hypochlorite (NaClO) solution is traditionally recommended for prion decontamination of equipment and surfaces. Using protein misfolding cyclic amplification with beads and a bioassay with TgElk mice, we compared the effects of these disinfectants in CWD-contaminated soil for 1 or 16 h to those of controls of known infectious titres. Our results suggest that 2 N NaOH in a 1/5 farm soil volume provides a large decrease (>10^2^-fold) in prion infectivity.

Chronic wasting disease (CWD) is a prion disease affecting cervids, which is now recognized in North America, Canada, Republic of Korea, Norway, and Finland [1–3]. Although the mechanism of CWD transmission is not well understood, there is evidence that infection is transmitted horizontally and can be acquired from environmental sources, indicating the importance of the shedding of infectious prions in the environment. The environment serves as a long-term reservoir of prion infectivity, which likely facilitates the sustained incidence of CWD in free-ranging cervid populations and complicates efforts to eliminate CWD and scrapie in captive herds [4]. Moreover, the current best practises for prion decontamination such as incineration and autoclaving are not practical for most environmental applications. Thus, development of a practical, *in situ* method for decontaminating soil and other environmental surfaces exposed to prions is highly desired; however, few effective decontamination techniques have been reported to date [5].

The disinfectants 2% NaClO and 2 N NaOH have been reported to inactivate conventional prion infectious agents and are recommended disinfectants by the OIE [6], which are highly corrosive but do not accumulate in the soil and are readily available for farmers working with CWD-contaminated soil. Thus, in the present study, we tested the effectiveness of these two chemical disinfectants using a bioassay and protein misfolding cyclic amplification with beads (PMCAb), which has proven to be a sensitive method to detect even low levels of prion infectivity [7–9].

The farm soil was collected from an elk-raising farm, which has a silt loam texture (Table 1). The CWD-contaminated soil was prepared from 28 g of farm soil (approximately 5 cm depth in a 50-mL conical tube). The farm soil was transferred to a plastic screw cap tube that was then irradiated by caesium-137 at a total dose 7.0 kGy, which is known to have the lowest effect on prion infectivity but inactivates bacteria and viruses [10]. For confirmation, the irradiated soil was tested on 5% sheep blood agar plates for residual microbial infectivity. The soil was saturated with distilled water and then 1% (w/v) natural CWD-infected elk brain homogenate was added at 3 mL per week for 4 months at room temperature. The infectivity titre of the CWD-infected elk brains was calculated to be 10^5.6^ LD_50_ per gram. The sample was then treated with the OIE-recommended disinfectants 2% NaClO and 2 N NaOH ranging from 0.1 (50 mL) to 1 (500 mL)-fold relative to the CWD-contaminated soil weight (0.5 g) for 1 and 16 h. Before the mouse bioassay and PMCAb were performed, the disinfected soil was washed eight times with phosphate buffered saline (pH 7.4) and spun in a slow rotator (Intellimixer, SEOLIN-BIO). Subsequently, 20 μL of a 14% soil suspension was collected and used for the bioassay and PMCAb.

**Table 1.** Physiochemical characteristics* of the farm soil.

pH	5.5
Soil texture	Silt loam
% clay	24.3
% sand	15.7
% silt	60
Organic matter, %	3.2
Total nitrogen, %	0.24
Cation exchange capacity (CEC), cmol/kg	20.2
Ca	3.8
Na	0.14
K	0.94
Mg	1.6

*All analyses were conducted at Korea Environmental Analysis Center.

For the bioassay, we used transgenic mice overexpressing elk prion protein (hereafter referred to as TgElk mice). This mouse line has been estimated to overexpress elk prion protein by 2.5 times [11]. All procedures involving mice in the current study were approved by the Animal Ethics Committee (AEC) of the Animal and Plant Quarantine Agency (APQA) under the Animal Protection Act of 1991 (Permit No: APQA-2016-449). The results with this bioassay showed that if 20 μL of untreated soil samples were inoculated intracranially without any inactivation treatment, the TgElk mice died after an average survival time of 130 days. Thus, for this study, six TgElk mice were inoculated intracranially with 20 μL of a given sample of disinfectants-treated soils. The inoculated mice were monitored twice a week and clinically assessed once a week for the following clinical parameters: markedly affected gait, generalized tremors and convulsions, rough coat, hunched back, and loss of weight and condition. When the animals were terminally ill, they were euthanized and necropsied. The whole brain was immediately frozen for western blotting (WB).

Disinfectant-untreated CWD-contaminated soil remained infectious up to a 100× dilution. Table 2 shows the effect of various amounts of the chemical treatments for 16 h on the CWD-contaminated soil. Mice inoculated with samples from the 2% NaClO solution (0.2:1) treatment group exhibited onset of the disease 222 days after inoculation. However, mice treated with samples subjected to the 2% NaClO solution (1:1) and all 2 N NaOH treatments did not show any signs of infection after more than 450 days.

**Table 2.** Attack rate and estimated reductions of disinfectants-treated soil samples in the TgElk mice bioassay.

Inoculum*	Attack rate	Survival time (dpi)**
Untreated (neat)	6/6 (100%)	134 ± 45
10× dilution	6/6 (100%)	207 ± 45
100× dilution	6/6 (100%)	261 ± 22
2 N NaOH (0.2:1)	0/6 (0%)	450
2 N NaOH (1:1)	0/6 (0%)	450
2% NaClO (0.2:1)	6/6 (100%)	261 ± 47
2% NaClO (1:1)	0/6 (0%)	450

*[volume proportion of disinfectant solution (mL) versus soil (g)]. ** dpi: day post-inoculation.

We previously examined the detection sensitivity of our amplification system for cervid CWD prions. Normal brain homogenate (NBH) was prepared by homogenization of TgElk brains with a glass dounce in nine volumes of cold PBS with TritonX-100, 5 mM EDTA, 150 mM NaCl, and 0.05% Digitonin (Sigma) plus Complete Mini Protease Inhibitors (Roche, Cat 1,836,145) to a final concentration of 5% (w/v). NBHs were centrifuged at 2,000 *g* for 1 min, and the supernatant was removed and frozen at −70°C until use. CWD-contaminated and disinfectant-treated soil was seeded in a 5% (w/v) NBH from 1:5, and then the next round samples were diluted at a 3:7 dilution ratio. Sonication was performed with a Misonix 4000 sonicator with the amplitude set to level 70, generating an average output of 160 W with two teflon beads (2.38 mm diameter, McMaster, Los Angeles, CA) per cycle. One round consisted of 56 cycles of 30 s of sonication followed by 9 min and 30 s of incubation 37°C. The samples (5 μL) obtained after each round of amplification were mixed with proteinase K (200 mg/mL) and incubated for 16 h at 37°C. Samples were separated by sodium dodecyl sulphate-polyacrylamide gel electrophoresis and transferred onto a polyvinylidene fluoride membrane. After blocking, the membrane was incubated for 1 h with the primary antibody S1 anti rabbit serum (APQA, 1:3000) and developed with an enhanced chemiluminescence detection system.

With the PMCAb reaction, PrP^CWD^ signals could be detected at a 10^−3^ dilution of the infected brain homogenate. However, the signal intensities were considerably weak in the first round. In the third round of amplification, PrP^CWD^ signals were detected in products diluted to 10^−9^ (data not shown). We could therefore confirm that the PMCAb method had extremely high detection sensitivity for cervid PrP^CWD^ with three rounds. Figure 1 illustrates the results of the amplification of the soil treated with the two kinds of chemical disinfectants. After three rounds of amplification, no signals were observed in the 2 N NaOH (0.2:1) and 2% NaClO (1:1)-treated soils for 1 or 16 h. However, treatment with 2% NaClO solution in 1/5 of the soil volume for 16 h (which resulted in death of infected mice in the bioassay) generated obvious PrP^CWD^ signals in the third round of amplification. These observations indicated that trace amounts of PrP^CWD^ had remained after these inactivation treatments. Taken together, the results obtained from three rounds of PMCAb were in good agreement with those of the bioassay.

**Figure 1.**
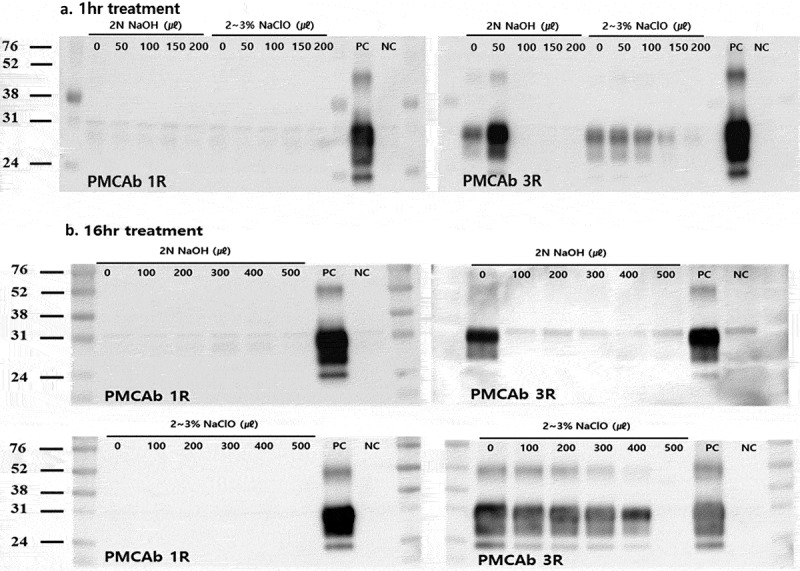
*In vitro* amplification of PrP^CWD^ in disinfectants-treated soil assessed by PMCAb. Different amounts of the recommended disinfectant concentrations (2 N NaOH, 2% NaClO) were treated to the soil for 1 and 16 h. (A) Western blot analysis after 1-h treatment. After three rounds of amplification, no signals were observed in the 100–200 μL 2 N NaOH-treated soils. In 2% NaClO-treated soils, PrP^CWD^ signals were detected for all amounts (50–200 μL). (B) Western blot analysis after 16-h treatment. After three rounds of amplification, no signals were observed in the 2 N NaOH-treated soils. PrP^CWD^ signals were not detected for only the 500 μL 2% NaClO-treated soils. Molecular mass standards (kDa) are indicated on the left. PC: positive control, 0.01% CWD infected elk brain homogenate; NC: negative control, 0.01% normal elk brain homogenate.

Complete decontamination of prion-contaminated tissues, surfaces, and environments can be difficult since prions are very resistant to most disinfectants, including formalin and alcohol, and few prion decontamination techniques have been developed and confirmed to be effective for routine use. New commercial disinfectants have been developed for prions, but their efficacies are highly variable [7]. The USDA also approved disinfectants that could be used in the field against foot-and-mouth disease, and the concentration was the same as that used for prion disinfection [12]. In Korea, CWD was only confirmed in elk in 2001, 2004, and 2005 [13]; however, additional cases were observed in red deer, sika deer, and their crossbred deer in 2010 and 2016 [14]. Therefore, it is important to prevent CWD recurrence in the Republic of Korea, and farmers that have experienced a CWD outbreak are required to disinfect the farm before reintroducing the cervids. Thus, farmers require a disinfectant solution that is marketed and readily available to effectively inactivate prions.

The prion titre in soil is correlated to the efficiency of *in vitro* amplification. Soil adsorption, which may protect PrP^Sc^ from environmental degradation or transport, is known to be greater for clay and organic surfaces and is lower for sand and sandy soils [15]. The soil used in this study is a silt loam, which is composed with clay (24.3%) and silt (60%) more than sand (15.7%) allowing highly containing of prion. These adsorption and disinfection were carried out for the small-scale cervid farm situation in Republic of Korea (average > 100 heads/farm). Our results from the detection of PrP^CWD^ amplified by three rounds of PMCAb agreed well with those from the TgElk bioassay. Silt loam soil-bound prion infectivity completely disappeared with treatment of 2 N NaOH for 16 h, which was more effective than treatment of 2% NaClO under the same condition. Although we only used a relatively small amount of soil, our results suggest that the use of 2 N NaOH at a 1/5 soil volume would result in a significant decrease in prion infectivity in farm soil.
